# Developing a core outcome set for chronic rhinosinusitis: a systematic review of outcomes utilised in the current literature

**DOI:** 10.1186/s13063-017-2060-y

**Published:** 2017-07-11

**Authors:** Archana Soni-Jaiswal, Raj Lakhani, Claire Hopkins

**Affiliations:** 1 Guys and St Thomas’s Hospital, London, UK; 2 George’s Hospital, London, UK; 3 King’s College, London, UK

**Keywords:** Rhinosinusitis, Patient outcomes assessment, Outcome measures

## Abstract

**Background:**

A core outcome set (COS) is an agreed standardised collection of outcomes that should be measured and reported by all trials for a specific clinical area, in this case chronic rhinosinusitis. These are not restrictive and researchers may continue to explore other outcomes alongside these that they feel are relevant to their intervention. The aim of this systematic review was to identify the need for a COS for chronic rhinosinusitis.

**Methods:**

A sensitive search strategy was used to identify all published Cochrane systematic reviews and randomised control trials of intervention for adult patients with chronic rhinosinusitis. Two independent authors reviewed these to obtain a list of outcomes and outcome measures reported by each clinical trial.

**Results:**

Sixty-nine randomised control trials and eight Cochrane systematic reviews were included in this study. They reported 68 individual outcomes and outcome measures, with an average of four to ten outcomes per clinical trial. These outcomes were mapped to 23 subcategories belonging to eight core categories.

**Conclusions:**

The key finding of this review was the heterogeneity of outcomes reported and measured by clinical trials of patients with chronic rhinosinusitis, precluding meaningful meta-analysis of data. This review supports the need for development of a COS, to be used in future trials on adult patients with chronic rhinosinusitis.

## Background

Chronic rhinosinusitis (CRS) is a disease in which patients develop inflammation of their nose and paranasal sinuses, with symptoms persistent for longer than 12 weeks. It may exist with or without nasal polyps [1]. It represents a common and widespread source of ill health in the UK with 11% of UK adults reporting symptoms of CRS [2]. It is also a source of substantial economic burden, through both direct health care costs and indirect societal costs secondary to lost economic productivity [3, 4].

Symptoms of CRS include nasal obstruction, nasal discharge, facial pain, loss of smell and sleep disturbance. When measured using the generic quality of life outcome measure, the Short-form-36, these symptoms have a major impact on patient’s quality of life, worse in some domains than other chronic diseases including angina and chronic respiratory disease [5]. Acute exacerbations, inadequate symptom control and respiratory disease exacerbation are common amongst this population. Complications are rare, but may include visual impairment and intracranial infection.

There is considerable variation in the way that CRS is managed. This relates, in part, to the lack of strong recommendations in treatment guidelines. There are a number of Cochrane reviews evaluating the effectiveness of treatments in CRS, but they are limited both by a paucity of high-quality randomised trials, and the heterogeneity of outcomes in those that have been reported which precludes meaningful meta-analysis. In order to overcome this, both the European Position Paper on Rhinosinusitis and Nasal Polyps, and the recently revised Cochrane systematic reviews, recognise the need to define a core outcome set for CRS [1, 6–10].

A COS is an agreed standardised collection of outcomes that should be measured and reported by all trials for a specific clinical area, in this case CRS [11]. The outcomes defined in a COS are not restrictive and trialists may continue to explore other outcomes alongside these that they feel are relevant to their intervention. However, the primary outcome should be one contained within the COS and if the COS is not being implemented then researchers should explain this decision in their findings [12].

The use of a COS in future trials of intervention in adult patients with CRS serves to minimise heterogeneity between outcomes reported by trials, allowing research data to be pooled for more meaningful meta-analysis, increasing both numbers of patients available and statistical power. The other advantage of a COS is that the outcomes are reflective of all health service users, including patients. The use of COS is supported by the World Health Organisation [13] and the Cochrane reviews of effects of health care interventions and have already been developed and adopted by multiple medical and surgical specialties [14].

The aim of this systematic review is to summarise the reporting standards of surgical outcomes in trials of intervention in adult patients with CRS with or without nasal polyposis, providing evidence in support of further development of a COS. It also aims to identify a list of outcomes required in the development of a COS.

## Methods

This systematic review was conducted in accordance with the published Preferred Reporting Items for Systematic Reviews and Meta-analysis (PRISMA) guidelines [15]. The study protocol was registered with the Core Outcome Measures in Effectiveness Trials (COMET) initiative (www.comet-initiative.org/) [16].

### Literature search

At the suggestion of COMET, high-quality randomised control trials for inclusion in this systematic review were obtained through a systematic search of the Cochrane Database of Systematic Reviews (16 Aug 2015) and the Cochrane Central Register of Controlled Trials (CENTRAL) (16 Aug 2015). The search was performed using the search terms, rhinosinusitis *or* ENT *or* Otolaryngology. Language, age and date restrictions were not applied to the searches.

### Inclusion criteria

Systematic reviews of trials of intervention in patients, 18 years or older, with CRS with or without nasal polyps were included. Those systematic reviews that had included patients with asthma, aspirin sensitivity and allergic fungal rhinosinusitis were also included.

### Exclusion criteria

Studies looking at CRS in a paediatric population, defined as age less than 18 years, studies of patients with allergic rhinitis or those with patients suffering with secondary CRS (patients with cystic fibrosis, granulomatosis with polyangiitis and other systemic diseases other than the ones listed above) were excluded. Published Cochrane systematic review protocols of on-going reviews were excluded.

### Study selection

The full-text articles for the Cochrane systematic reviews were obtained and analysed for eligibility by two independent reviewers, with a third reviewer available to adjudicate discrepancies.

As each systematic review presents the results of a number of pooled clinical trials, the published papers for each individual trial were obtained. The bibliographies of the trial papers were further evaluated to identify additional studies for inclusion, not included within the Cochrane systematic reviews themselves. If we could not find the published full-text article of each randomised control trial, the authors were contacted directly to see if they could provide a copy of their original research paper or thesis.

### Data extraction

Two independent reviewers extracted data from the randomised controlled trials (RCTs). Two independent spreadsheets were created and the primary outcome (if defined), secondary outcome and the Outcome Measure Instrument (OMI) used to measure both the primary and secondary outcome in each trial listed. These outcomes and outcome measures were obtained from the method and results section of each paper. If the paper did not explicitly mention the outcomes that they measured, then the reviewer inferred these from the given data. At the end of the data extraction process, a final list of outcomes was compiled by consensus between both reviewers and a single spreadsheet created, containing details of each study, including sample size, study type, outcome and validity of outcome measure.

Previous work perfumed as part of the OMIPP project, commissioned by Cochrane UK, surveyed Public, Patients and Practitioners to identify a list of outcomes that they felt were important in Cochrane reviews and trials of intervention for patients with CRS. We employed the categories identified as part of this project, using them to map our outcomes and OMIs, as appropriate [17]. The OMIPP project identified 23 predetermined core categories belonging to eight main categories, namely ‘Changes in patient-related symptom severity’, ‘Quality of life’, ‘Physiological assessments’, ‘Microbiological’, ‘Biomarkers’, ‘Lower airway disease’, ‘Side effects’ and ‘Acceptability of treatment’.

Each individual outcome from this study was mapped to a core subcategory. These subcategories were then grouped into main categories which were then placed into the Outcome Measures in Rheumatoid Arthritis Clinical Trials (OMERACT) filter 2.0. The OMERACT filter 2.0 has been developed by a group of international health care professionals involved in improving outcomes reporting in rheumatology and one of the first groups to develop a COS. They have provided a framework to be considered when developing a COS which includes death, life impact and resource use/emotional impact, pathophysiological manifestations and adverse events [18].

## Results

**Fig. 1:**
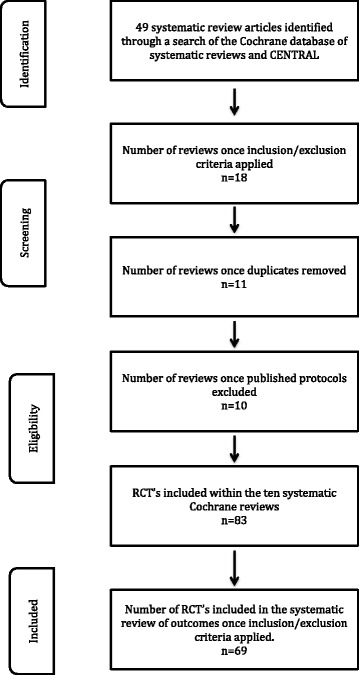
Preferred Reporting Items for Systematic Reviews and Meta-analysis (PRISMA) flowchart reflecting the review methodology

Following a systematic search of the Cochrane database, using the PRISMA guidelines, a total of 49 systematic Cochrane review articles were identified Once the inclusion and exclusion criteria were applied, this number reduced to 18. Duplicate entries and published protocols of on-going studies were excluded to leave ten systematic reviews which were included in this study. A summary flowchart is provided in Fig. 1 below.

A total of 83 RCT papers were included within these ten Cochrane systematic review papers. Of these, 14 were excluded as they were trials of paediatric patients, those with allergic rhinitis or published conference abstracts. A total of 69 RCTs were finally obtained from these systematic reviews and included within this study. No further references were identified through examination of the bibliography of the published systematic reviews or trials. All included articles were published in the English language. The included clinical trials were published between the years 1975 and 2012. Table 1 provides the details of the systematic reviews and the number of trials extracted from each of these.

**Table 1:** Summary of included Cochrane systematic reviews

Cochrane systematic review	Number of RCTs included within the systematic review	Number of RCTs that did not fit our study protocol	Total number of RCTs included from the systematic review in our study
Khalil H, Nunez DA. Functional endoscopic sinus surgery for chronic rhinosinusitis (2006, updated in 2009) [21]	3	0	3
Harvey R et al. Nasal saline irrigations for the symptoms of chronic rhinosinusitis (2007) [22]	8	5	3
Piromchai P et al. Systemic antibiotics for chronic rhinosinusitis without nasal polyps in adults (2011) [23]	1	0	1
Sacks PL et al. Topical and systemic antifungal therapy for the treatment of chronic rhinosinusitis (2011) [24]	6	0	6
Snidvongs K et al. Topical steroids for chronic rhinosinusitis without nasal polyps (2011) [25]	11	1	10
Ahmed J et al. Functional endoscopic balloon dilation of sinus ostia for chronic rhinosinusitis (2011) [26]	2	1	1
Kalish L et al. Topical steroids for nasal polyps (2012) [27]	44	7	37
Rimmer J et al. Surgical versus medical interventions for chronic rhinosinusitis with nasal polyposis (2014) [20]	8	0	8
Huang Z et al. Steroid-eluting sinus stents for improving symptoms in chronic rhinosinusitis patients undergoing functional endoscopic sinus surgery (2015) [28]	0	0	0
Sharma R et al. Surgical interventions for chronic rhinosinusitis with nasal polyps (Review) (2014) [29]	0	0	0

Three hundred and sixty-five individual outcomes were extracted from the clinical trials. The trials overlapped in their use of outcomes and between them had used 68 different outcomes and OMIs. Initially these were divided into two broad categories, namely category 1 ‘outcome’ and category 2 ‘Outcome Measure Instrument (OMI) used to assess the specific outcome’. This produced an extensive list, with multiple OMIs used to measure individual outcomes (Table 2). We employed the categories identified as part of the OMIPP project (previously discussed in the ‘Methods’ section), using them to map our outcomes and OMIs, as appropriate [17]. Hence, our long-list was mapped onto 23 predetermined ‘core categories’, belonging to eight ‘main categories’, namely ‘Changes in patient-related symptom severity’, ‘Quality of life’, ‘Physiological assessments’, ‘Microbiological’, ‘Biomarkers’, ‘Lower airway disease’, ‘Side effects’ and ‘Acceptability of treatment’ (Table 3).

**Table 2:** A long-list of reported outcomes and Outcome Measure Instruments (OMIs) extracted from the trials

Outcome measure	Adjunctive OMIs used to measures this across the trials
Patient history of CRS: nasal blockage/congestion, nasal discharge, olfactory disturbance, facial pressure or pain, headache	Visual Analogue Scale (VAS) – multiple variations, SNOT-20, modified RSDI, SNOT-22, RSOM-31, Chronic Sinusitis Survey, SNAQ-11
Overall discomfort	SNOT-20, Short-form-36 (SF-36)
Adjunctive symptoms of sneezing, itching, cough, Eustachian tube discomfort, dizziness, otalgia	SNOT-20, SNOT-22
Impact on quality of life with inclusion of mental health, sleep disruption, fatigue, productivity, concentration	SNOT-20, SNOT-22, SF-36, Short-form-12 (SF-12)
Nasal swelling and local or systemic inflammation	Acoustic rhinomanometry, nitric oxide (NO), peak nasal inspiratory flow (PNIF), nasal mucosal eosinophilia on nasal smear or biopsy, intranasal inflammatory markers such as interleukins, serum inflammatory markers including WCC and differential, ESR, CRP, serum immunoglobulins with emphasis on IgE, skin-prick testing and serum RAST testing
Altered mucociliary function	Saccharine clearance test
Surgical and medical complications	Patient history, patient diary, blood tests for steroid-induced side effects, ocular assessments including slit-lamp examination, tonometry studies, visual acuity, colour vision charts, oral swabs for candidiasis
Intranasal polyps, discharge, oedema, adhesions, crusting	Anterior rhinoscopy, nasal endoscopy, polyp histology
Treatment compliance, adjunctive use of rescue medication, comfort of treatment	Patient diary, history, number of bottles or volume of spray/inhaler used, number of empty douche bottles
Extent of sinonasal disease	Nasal endoscopy, CT scan (Lund-Mackay or Catalano and Payne modification of the frontal recess), plain sinus X-ray, MRI
Presence of intranasal bacteria and fungi	Swab/secretion microscopy and culture, intranasal fungal protein (*Alternaria*) levels
Impact on asthma	History, global initiative for asthma guidelines, patient diary, VAS, peak expiratory flow rate, FEV_1_, pulmonary function tests, exhaled respiratory NO
Olfaction	History, VAS, SNOT-20, SNOT-22, butanol threshold test, University of Pennsylvania Smell Identification Test (UPSIT), coffee turpentine and lavender oil smell assessment on a scale of 0–3, oflactometry, Sniffin Sticks
Length of improvement with treatment	X-rays, history

*CRP* C-reactive protein, *CT* computed tomography, *ESR* erythrocyte sedimentation rate, *FEV* forced expiratory volume, *MRI* magnetic resonance imaging, *NO* nitric oxide, *RSDI* Rhinosinusitis Disability Index*, RSOM* Rhinosinusitis Outcome Measure*, SNAQ* Sino-nasal Questionnaire*, SNOT* Sino-nasal Outcome Test*, WCC* white cell count

**Table 3:** Exhaustive list of all reported outcomes/outcome measures mapped onto the OMIPP ‘core categories’ and ‘main categories’

OMIPP ‘main category’ (8)	OMIPP ‘core category’ (23)	Specific outcome/Outcome Measure Instrument used by trials (as outlined more extensively in Table 2)
Changes in patient-rated symptom severity	Global	VAS (multiple different nonvalidated scales used), patient diary
	Disease-specific	VAS for nasal blockage/congestion, nasal discharge, olfactory disturbance, facial pain/pressure, headache, itching, sneezing, ocular symptoms. Clinical history for snoring, rhinitis, nasal obstruction, headache, dry mouth, loss of smell, use of medication
Quality of life	Global	SF-36, SF-12, 7-point scale
	Disease-specific	SNOT-20/21/22, RSDI, RSOM-31, SNAQ-11, Chronic Sinusitis Survey
Physiological assessments	Endoscopic	VAS for adhesions, stenosis of ostia, patency of frontal recess, blood crusts, turbinate size. Polyp size using the Malm, Lindholt or Lund-Mckay scores. Descriptive text about mucosal oedema, discharge, crusting, scarring and swelling
	Radiological	CT using Lund-Mckay scores or Catalano and Payne for the frontal recess, MRI, X-ray
	Nasal airflow	PNIF, PNEF
	Mucociliary Function	Saccharine clearance testing
	Olfactory testing	VAS scores, butanol threshold testing, carbinol sniff bottles, UPSIT, individual odours such as coffee/turpentine and lavender
	Sinus manometry	Active anterior rhinomanometry, acoustic anterior and posterior rhinomanometry
Microbiological	Microbiome	Antral fluid culture, lavage culture, nasal swabs, *Alternaria* protein levels, middle meatus swab, nasal discharge swab
	Fungal hyphae	As above
Biomarkers		NO, IL-8, IL-5, IL- 4, fuxose, serum eosinophils, mucous eosinophil-derived neurostosin, α-2 macroglobulin, IL-lβ, TNFα in nasal lavage, mucosal biopsy for CD4, IL4, MBP and T-cells. Skin allergy testing, serum IgE, ESR, WCC, CRP
Lower airway disease	Patient-reported outcome measures	Not assessed by any trial
	Patient symptoms	VAS for SOB, cough, wheeze, breathlessness, need for a B_2_-agonist
	Respiratory function tests	FEV_1_, FVC, VC, MEF50, histamine inhalation challenge, PEFR, exhaled NO
Side effects	Medical	Patient diary, epistaxis rates, plasma cortisol levels, serum ACTH, ophthalmological ocular assessment, urinary cortisol levels, oral candidiasis
	Surgical	Epistaxis, synechia, sinonasal infections, revision surgery
Acceptability of treatment	Compliance	VAS, patient diary, empty medicine containers
	Acceptability to patients	Discomfort, length of improvement
	Cost incurred by patient	Not assessed by any trial
	Cost to third party	Not assessed by any trial
	Patient preference	Based on history from patient in only one RCT
Other		Need for rescue medication, rates of revision surgery

*ACTH* adrenocorticotropic hormone, *CRP* C-reactive protein*, CT* computed tomography*, ESR* erythrocyte sedimentation rate*, FEV* forced expiratory volume*, FVC* forced vital capacity*, IL* interleukin*, MEF50* maximal expiratory flow at 50%, *MBP*, major basic protein *MRI* magnetic resonance imaging*, NO* nitric oxide*, PEFR* peak expiratory flow rate*, PNEF*, peak nasal expiratory flow *PNIF*, peak nasal inspiratory flow *RCT* randomised controlled trial, *RSDI* Rhinosinusitis Disability Index, *RSOM* Rhinosinusitis Outcome Measure*, SF* Short-form*, SNAQ* Sinonasal Questionnaire*, SNOT* Sino-nasal Outcome Test*, SOB* shortness of breath*, TNF* tumour necrosis factor, *VC* vital capacity, *WCC* white cell count

These main categories were then mapped into the five OMERACT subheadings, ‘Life Impact’, ‘Pathophysiology’, ‘Resource use/Economic impact’, ‘Adverse Events’ and ‘Death’ (Table 4). No clinical trial in our study had included an outcome for the OMERACT core area ‘Death’ [18].

**Table 4:** Mapping of main categories and core categories into the Outcome Measures in Rheumatoid Arthritis Clinical Trials (OMERACT) headings

OMERACT heading	Life impact		Pathophysiology		Resource use/economic impact		Adverse events		Death
1	Patient subjective perception of health and severity of symptoms	Number of trials to use the outcome	Physiological assessments	Number of trials to use the outcome	Cost	Number of trials to use the outcome	Medical	Number of trials to use the outcome	Not reported by any clinical trials
	a. Global	13	a. Endoscopic	55	a. Personal	0		32	
	b. Disease-specific	51 (measured primarily with the Visual Analogue Score (VAS)	b. Radiological	25	b. Societal	0			
	c. Individual Lower airways	4	c. Nasal airflow	26					
			d. Mucociliary function	7					
			e. Olfactory testing	10					
			f. Sinus manometry	11					
			g. Respiratory function test	5					
2	Quality of life		Microbiological	11	Need for rescue medications	10	Surgical	1	
	a. Global	10 (most common outcome measure used was the SF-36)							
	b. Disease-specific	13 (SNOT, RSDI, RSOM 31)							
3	Resource utilisation		Biomarkers	15	Need for revision surgery	1			
	a. Compliance	7							
	b. Acceptability	2							
	c. Patient preference	1							

*RSDI* Rhinosinusitis Disability Index, *RSOM* Rhinosinusitis Outcome Measure, *SNOT* Sino-nasal Outcome Test

Most trials reported between four and ten outcomes each. The most frequently reported outcome was the patient’s individual perception of their health and severity, measured using either a Visual Analogue Scale (VAS) or the patient’s history or patient diary (*n* = 51 RCTs). The most frequently used outcome measure was an endoscopic assessment of the patient’s sinonasal cavity (*n* = 55 RCTs).

## Discussion

The COMET initiative was supportive of this study and, at the recommendation of their lead biostatistician, we extracted outcomes from RCTs that had been included in Cochrane systematic reviews, hopefully reflecting high-quality, well-designed research studies.

The key finding of this systematic review was the heterogeneity of outcomes reported and measured by Cochrane systematic reviews and clinical trials of adult patients with CRS. Most trials had not explicitly defined the primary outcome for their study or provided definitions for the outcomes that they were measuring, with many simply providing a list of outcome measures, such as nasoendoscopy or radiology, that were being used by them to evaluate effectiveness of treatment in their study. Sixty-nine RCTs had employed 68 individual outcomes between them to report effectiveness of various treatments being studied on patients with CRS. The biggest drawback of this is the inability to pool patient data and perform meaningful meta-analysis.

A positive finding was that most studies had included a measure of ‘life impact’ reflecting the patient’s perception of their own health and its impact on their quality of life. However this was achieved using a variety of VASs or simply based on the patients history, hence making comparison between studies more difficult.

The most commonly reported subgroup of outcomes was ‘Pathophysiology’. Some of the outcomes included in this group were the outcome measures ‘nasoendoscopy’, ‘radiology’, ‘mucociliary function’, ‘nasal volume’ and ‘resistance’. This is unsurprising as these are frequently used clinical measures that allow clinicians to assess objective response to treatment. However, 16 different outcomes measures had been used in this category alone, hence once again making direct comparison between studies difficult.

None of the included studies had assessed ‘resource use/economic impact’ of disease and only a handful had asked about patient preference and acceptability of the treatment to the patient. These may be of importance when considering compliance and longevity of treatment. They may also be important to stakeholders other than physicians, including patients and third parties such as health care providers, insurance companies and drug companies.

The OMERACT category ‘death’ was not reported by any of our trials. Patients with CRS are generally fit patients undergoing elective surgery or medical therapy and hence we would not expect that patients should die as a direct result of the treatment being offered [18]. We must remember that the OMERACT filter 2.0 was developed for patients with rheumatoid arthritis, and although the breadth of outcomes it offers is excellent, it must perhaps be modified for our cohort of patients with CRS, with the exclusion of the death category.

We found that historical trials reported fewer outcomes, rarely defined the outcome and used simpler OMIs such as nonvalidated VAS scores and facial roentgenograms. After the late nineties these evolved to include better-defined outcomes, with multiple validated outcome measures. Also, the trend for inclusion of qualitative outcome measures changed with time; with newer studies including patient-reported outcome measures alongside qualitative outcomes.

The key strength of this study is the large number of clinical trials included, providing an exhaustive list of outcomes that have been reported in the academic literature. However, a limitation of this study is that we have only included outcomes that are important to researchers and clinicians. We have not captured outcomes important to patients themselves or other stakeholders such as primary care physicians.

To improve the quality of trials included in this review, with minimal bias, only those studies published within Cochrane systematic reviews were included in this study. Hence, a study of other search engines, as would be routine practice when researching a systematic review, was not performed. This does risk missing some good trials published independently of the Cochrane reviews.

In order to overcome the two limitations discussed, the outcomes obtained through this review, will be combined with (a) outcomes obtained through patient focus groups, (b) outcomes obtained through Public, Patient and Practitioner surveys [17] and (c) outcomes obtained through interviews with family practitioners and otolaryngologists. It is this final long-list of outcomes, representing all stakeholders, that will be used in the development of a COS via the Delphi process [12, 19].

The Cochrane Collaboration has recently updated a few of the published systematic reviews included in this study [6–8, 10, 20]^.^ For completeness, full-text articles of these updated reviews and any trials not previously reviewed were analysed. This has not revealed any adjunctive outcomes, for inclusion in the COS development process.

Once a final list of outcomes has been formulated, we will ask patients and clinicians to define the outcomes that have been extracted from these trials, providing standardised definitions that we, both clinicians and patients, understand. The involvement of patients in contributing and then defining outcomes will ensure that the target audience for the treatment is an active participant in the development of the COS.

As previously explored, developing a COS for this subgroup of patients will allow meaningful comparisons to be made between future clinical trials of novel and existent therapies, with the ability to combine results and produce meaningful, high-quality research data, with an overall positive impact on patient management.

## Conclusion

This systematic review supports the need for development of a COS in CRS and has identified a potential list of outcomes to be used in this process.

## Abbreviations

COMET: Core Outcome Measures in Effectiveness Trials
COS: Core outcome set
CRS: Chronic rhinosinusitis
OMERACT: Outcome Measures in Rheumatoid Arthritis Clinical Trials
PRISMA: Preferred Reporting Items for Systematic Reviews and Meta-analysis
RCT: Randomised control trial
